# HDAC1-mediated regulation of GABA signaling within the lateral septum facilitates long-lasting social fear extinction in male mice

**DOI:** 10.1038/s41398-023-02310-y

**Published:** 2023-01-17

**Authors:** Anna Bludau, Inga D. Neumann, Rohit Menon

**Affiliations:** Department of Behavioural and Molecular Neurobiology, Regensburg Center of Neuroscience, University of Regensburg, Regensburg, Germany

**Keywords:** Long-term memory, Epigenetics and behaviour, Molecular neuroscience

## Abstract

Social anxiety disorder (SAD) is caused by traumatic social experiences. It is characterized by intense fear and avoidance of social contexts, which can be robustly mimicked by the social fear conditioning (SFC) paradigm. The extinction phase of the SFC paradigm is akin to exposure therapy for SAD and requires learning to disassociate the trauma with the social context. Learning-induced acetylation of histones is critical for extinction memory formation and its endurance. Although class I histone deacetylases (HDACs) regulate the abovementioned learning process, there is a lack of clarity in isoforms and spatial specificity in HDAC function in social learning. Utilizing the SFC paradigm, we functionally characterized the role of HDAC1, specifically in the lateral septum (LS), in regulating the formation of long-term social fear extinction memory. We measured a local increase in activity-inducing HDAC1 phosphorylation at serine residues of social fear-conditioned (SFC^+^) mice in response to the extinction of social fear. We also found that LS-HDAC1 function negatively correlates with acute social fear extinction learning using pharmacological and viral approaches. Further, inhibition of LS-HDAC1 enhanced the expression of the GABA-A receptor β1 subunit (*Gabrb1*) in SFC^+^ mice, and activation of GABA-A receptors facilitated acute extinction learning. Finally, the facilitation of extinction learning by HDAC1 inhibition or GABA-A receptor activation within the LS led to the formation of long-lasting extinction memory, which persisted even 30 days after extinction. Our results show that HDAC1-mediated regulation of GABA signaling in the LS is crucial for the formation of long-lasting social fear extinction memory.

## Introduction

Social anxiety disorder (SAD) caused by aversive social experience is characterized by intense fear and avoidance of social situations [1, 2]. Treatment for SAD consists of exposure-based cognitive-behavioral therapy (CBT), which creates a “safety” memory that co-exists with the original aversive memory [3, 4]. Since the same cue can activate both aversive and safety memories, they compete when recalled by relevant stimuli, and the dominant memory trace determines the behavioral outcome. The safety memory created by exposure-based CBT is known to diminish over time, leading to a relapse of previously extinguished fear responses in SAD patients [5–7]. Thus, it is imperative to understand the mechanisms underlying enduring extinction memory to develop therapeutic strategies that lead to more complete and robust remission from SAD.

To this end, we established the operant social fear conditioning (SFC) paradigm, which generates robust fear and avoidance of conspecifics in mice [8]. In this paradigm, social fear extinction and formation of extinction memory are induced by consecutive presentations of different social stimuli (unknown conspecifics), resulting in a gradual loss of fear and increased social investigation [9]. Previous studies from our lab have shown the involvement of the lateral septum (LS) in the extinction of SFC-induced social fear in mice [10, 11]. The LS is also known to regulate various social behaviors [12], which are underpinned by complex epigenetic mechanisms [13]. However, the epigenetic mechanisms within the LS regulating the extinction of social fear and the formation of long-term extinction memory remain unknown.

Concurring evidence suggests the importance of post-translational acetylation of N-terminal tails of histone residues in the consolidation and maintenance of fear memory [14]. Class I histone deacetylases (HDACs), which catalyze the removal of acetyl groups from these N-terminal tails of histones, are essential regulators of gene expression underlying learning and memory [15]. These class I HDACs, including HDAC1, HDAC2, HDAC3, and HDAC8, show remarkable spatial and contextual dependency in their function. For instance, hippocampal HDAC1 was found to enhance associative memory in a contextual fear conditioning paradigm [16], while hippocampal HDAC2 has been functionally linked to impairment in long-term object and spatial memory formation [17, 18]. Similarly, HDAC1 activity was upregulated, while HDAC2 activity was downregulated within the amygdala and the prefrontal cortex, respectively, following cued fear conditioning [19]. Studies have also implicated the differential contribution of HDAC3 in the formation of robust aversive contextual and cued fear memories in the hippocampus and basal amygdala, respectively [20]. Considering the varied and complicated functions of class I HDACs in the regulation of learning and memory, HDAC inhibitors are often used as cognitive enhancers in several pre-clinical studies [21]. However, the contribution of specific HDACs in regulating the endurance of social fear extinction memory remains elusive.

Thus, in this study, we examined the regulation of class I HDACs in the context of the SFC paradigm. Based on the post-extinction increase in activity-inducing serine phosphorylation of HDAC1, we manipulated LS-HDAC1 function using pharmacological inhibition and viral overexpression. Furthermore, following up on the increase in the expression of the gene coding for the β1 subunit of the gamma-aminobutyric acid (GABA) A receptor (*Gabrb1*) in response to the combination of HDAC1 blockade and extinction training, we activated GABA-A receptor signaling within the LS. Finally, we tested for the effect of LS-HDAC1 inhibition and LS-GABA-A receptor activation on long-term extinction memory formation. Our results indicate that LS-HDAC1 negatively regulates social fear extinction learning and long-term social fear extinction learning via GABA-A signaling.

## Materials and methods

### Animals

Male CD1 mice (Charles River Germany, 8–11 weeks of age at the start of experiments) were kept group-housed under standard laboratory conditions (12/12 h light/dark cycle, lights on at 06:00, 22 °C, 60% humidity, food and water *ad libitum*) in polycarbonate cages (16 × 22 × 14 cm) until surgery or 3 days before each behavioral experiment. Age and weight-matched male CD1 mice were used as social stimuli in the SFC paradigm. All experimental procedures were performed between 08:00 and 12:00 h in accordance with the Guide for the Care and Use of Laboratory Animals of the Government of Unterfranken, ARRIVE guidelines [22], and the guidelines of the NIH.

### SFC paradigm

SFC was performed as previously described [8] with minor modifications for analyzing long-term extinction:

#### Social fear acquisition (day 1)

Mice were placed in the fear conditioning chamber and allowed to habituate for 30 s before they were exposed to an empty wire-mesh cage for 3 min during which they could freely investigate the object. Then the empty cage was replaced with an identical cage containing age and weight-matched conspecific (conditioned stimulus: CS). Conditioned mice (SFC^+^) received a mild foot shock (unconditioned stimulus: US; 0.7 mA, 1 s) each time they investigated the conspecific. Unconditioned mice (SFC^**−**^) were allowed to freely interact with the conspecific for 3 min. Usually, SFC^+^ mice avoided approaching or interacting with the conspecific after 1–3 CS-US pairings, at which point they were considered to have acquired social fear and returned to their home cage.

#### Social fear extinction (day 2)

Social fear extinction was performed identically in the home cage of SFC^+^ and SFC^−^ mice. Each single-housed mouse was exposed three times to an empty wire-mesh cage, then to six different age and weight-matched conspecifics for 3 min each, with a 3-min interstimulus interval. The time spent investigating the social stimulus was measured as an indicator of social fear.

#### Social fear recall (day 3, day 32)

For short-term social fear extinction recall, mice were exposed to six different social stimuli in their home cage for 3 min with a 3-min interstimulus interval. For the long-term social fear extinction recall, mice were tested in their home cage 30 days after social fear extinction, wherein they were exposed twice to different social stimuli for 3 min each with a 3-min interstimulus interval.

### Stereotaxic implantations

Guide cannulas (23 G, 8 mm length; Injecta GmbH) for bilateral drug infusion into the LS were implanted stereotaxically with the tip resting 2 mm above the LS target region (from bregma: anterior–posterior: −0.3 mm, mediolateral: ±0.5 mm, dorsoventral: −1.6 mm [23]), under isoflurane anesthesia (Forene, Abbott GmbH) [10, 24]. All mice received a subcutaneous injection of antibiotics (10 mg/kg Baytril, Bayer GmbH) and analgesics (0.05 mg/kg Buprenorphine, Bayer) to avoid post-surgical infections and pain. In order to reduce post-surgical stress, all mice were handled once a day for at least 5 days before experiments wherein dummy cannulas closing the guide cannulas were disinfected.

### Intraseptal drug infusions

Mice received bilateral infusions of either MS275 (an *ortho-*amino anilide-based small molecule inhibitor with high affinity for HDAC1; 375 ng/0.2 µl/hemisphere) [16, 25], muscimol (Muc; a selective GABA-A receptor agonist; 0.2 µg/0.2 µl/hemisphere) [26, 27] or Vehicle (Veh; 0.2 µl/hemisphere; 0.5% DMSO in sterile Ringer) into the LS via a 27 G infusion cannula inserted into the guide cannulas connected to a 10-µl Hamilton syringe. Infusion of MS275 and Muc was performed 120 min or 10 min prior to SFC extinction training, respectively. Only animals with correct, histologically verified infusion sites were included in the statistical analyses.

### Intraseptal viral infusions

The Adeno-associated virus (AAV; Vector Biolabs) for overexpression of HDAC1, i.e., AAV-hSyn-HDAC1-GFP-WPRE (HDAC1-OE; 2.5 × 10^13^ GC/ml;), or the control vector, i.e., AAV-hSyn-GFP-WPRE (GFP; 2.5 × 10^13^ GC/ml), were bilaterally infused into the LS (anterior–posterior from bregma +0.3 mm, lateral ±0.5 mm; dorsoventral −3.8 mm, −3.4 mm, −3.1 mm, −2.8 mm) with four 70-nl infusions/hemisphere. Infusions were performed 3 weeks prior to the start of behavioral experiments as previously described [11].

### Gene expression analysis

Mice were subjected to social fear extinction and left undisturbed in their home cages for 90 min after behavioral training. Mice were then sacrificed under CO_2_ anesthesia, their brains were rapidly removed, and the septum (anterior–posterior from bregma 0.1 mm to −0.10 mm) was dissected.

### RNA isolation and PCR array

Total RNA was isolated using peqGOLD TriFast (VWR, Radnor, USA) according to the manufacturer’s protocol. The RT^2^ Profiler PCR Array GABA & Glutamate (Qiagen) was used to identify altered mRNAs according to the manufacturer’s protocol. mRNAs were normalized against the geometric mean of five housekeeping genes. A list of housekeepers can be found in Supplementary Table S1.

### Protein extraction and analysis

Nuclear proteins were extracted using the EpiQuick Nuclear Extraction Kit (Epigentek) according to the manufacturer’s protocol. Western blot analysis was used to detect HDAC1 expression and phosphorylation levels. 15 μg of protein were resolved onto 12% TGX Stain-Free Gels (Bio-Rad) and transferred to nitrocellulose membranes. Membranes were blocked with 5% milk, 1% Tween-20 in Tris-buffered saline followed by overnight incubation with the following antibodies: rabbit-anti-HDAC1 (Thermo Fisher; PA1-860; 1:2000), HDAC1 phosphorylation on serine residue 421 (rabbit-anti-pHDAC1(S421); Thermo Fisher; PA5-36810; 1:2000), as well as phosphorylation on serine 421 and 423 (rabbit-anti-pHDAC1(S421, S423), Thermo Fisher; PA5-36911; 1:2000). Immunolabeled bands were detected using the HRP-conjugated goat-anti-rabbit antibody (Cell Signaling; #7074; 1:1000) following an enhanced chemiluminescence system (SuperSignal West Dura, Thermo Fisher). Total protein was used as an internal loading control.

### Scoring of behavior and statistical analysis

Social investigation times as indicator of social fear were manually scored by an observer blind to treatment using JWatcher (1.0, Macquarie University and UCLA). GraphPad Prism version 9.0 for Windows (GraphPad Software) was used for statistical analysis. A detailed report for all statistical analyses is available in Supplementary Table S2. All figures were produced using Affinity design.

## Results

### Social fear extinction upregulates activity-inducing HDAC1 phosphorylations in the septum of SFC^+^ mice

**Fig. 1:**
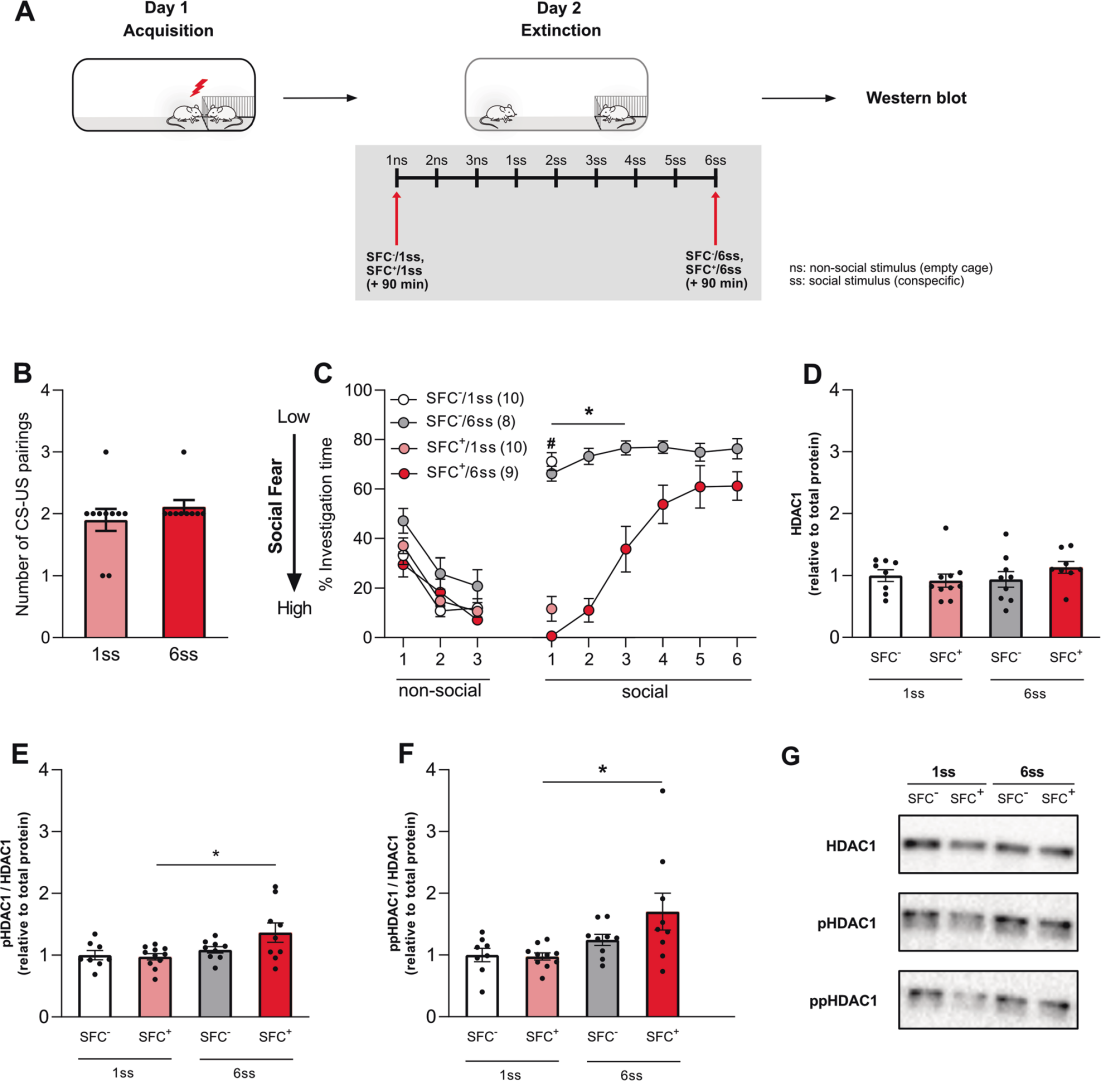
Phosphorylation on serine residues of septal HDAC1 is upregulated in response to social fear extinction. Schematic representation of the experimental timeline wherein red arrows indicate the time point of sacrifice (**A**). Mice were fear conditioned (SFC^+^) or left unconditioned (SFC^−^) on day 1 of the SFC paradigm. The number of CS-US pairings for groups of SFC^+^ mice exposed to one social stimulus (1ss) and six social stimuli (6ss) during extinction (**B**). Total protein was isolated from the septum 90 min either after abridged social fear extinction (SFC^−^/1ss and SFC^+^/1ss *n* = 10/group) or complete extinction (SFC^**−**^/6ss and SFC^**+**^/6ss; *n* = 8–9/group; **C**). Relative protein levels of HDAC1 (**D**), pHDAC1 (Ser421) (**E**), and ppHDAC (Ser421, Ser423) (**F**) were measured by western blot. Representative western blot images for HDAC1, pHDAC1, and ppHDAC1 (**G**). Data represent mean CS-US pairings ± SEM (**B**), mean investigation time ± SEM (**C**), or fold change + SEM (**D**–**F**). *P < 0.05 SFC^−^/6ss vs. SFC^+^/6ss and ^#^*P* < 0.05 SFC^−^/1ss vs. SFC^+^/1ss (**C**) or between indicated groups (**E**, **F**).

First, we analyzed HDAC1 protein levels in septal tissue micropunches obtained from SFC^+^ and SFC^−^ mice 90 min after exposure to either only one social stimulus, i.e., with abridged extinction (SFC^+^/1ss and SFC^−^/1ss), or six social stimuli, i.e., with complete extinction (SFC^+^/6ss and SFC^−^/6ss) (Fig. 1A). The number of CS-US pairings during social fear acquisition on day 1 (Fig. 1B) was similar between both groups of SFC^+^ mice. As expected, SFC^+^ mice spent significantly less time investigating the first (or following) social stimuli compared to SFC^−^ mice (*P* < 0.05; Fig. 1C), indicating higher levels of social fear. However, by the end of the complete extinction protocol, the investigation time of the 6th social stimulus by SFC^+^/6ss mice was similar to that of their SFC^−^/6ss counterparts (Fig. 1C), suggesting successful social fear extinction.

Western blot analysis did not reveal differences in septal HDAC1 levels between the four groups tested, i.e., between SFC^+^/1ss, SFC^+^/6ss, SFC^−^/1ss, and SFC^−^/6ss, during extinction of social fear (Fig. 1D). Post-translational phosphorylation of HDAC1 on ser421 (pHDAC1) and double-phosphorylation at ser421 and ser423 (ppHDAC1) are known to increase the deacetylase activity of HDAC1 [28]. Thus, we quantified pHDAC1 and ppHDAC1 levels within the septum to assess the activation of HDAC1 during social fear extinction. Both pHDAC1 and ppHDAC1 levels were increased in SFC^+^/6ss compared to SFC^+^/1ss mice (Fig. 1E; *P* < 0.05 for pHDAC1 and Fig. 1F; *P* < 0.05 for ppHDAC1), suggesting an increase in HDAC1 activity during extinction of social fear.

### Bidirectional modulation of LS-HDAC1 regulates learning of social fear extinction

Next, we aimed to establish a causal relationship between LS-HDAC1 function and social fear extinction by bidirectional modulation of LS-HDAC1 function using either pharmacological blockade (loss of function) or an AAV-mediated overexpression (gain of function) in two separate cohorts of mice.

**Fig. 2:**
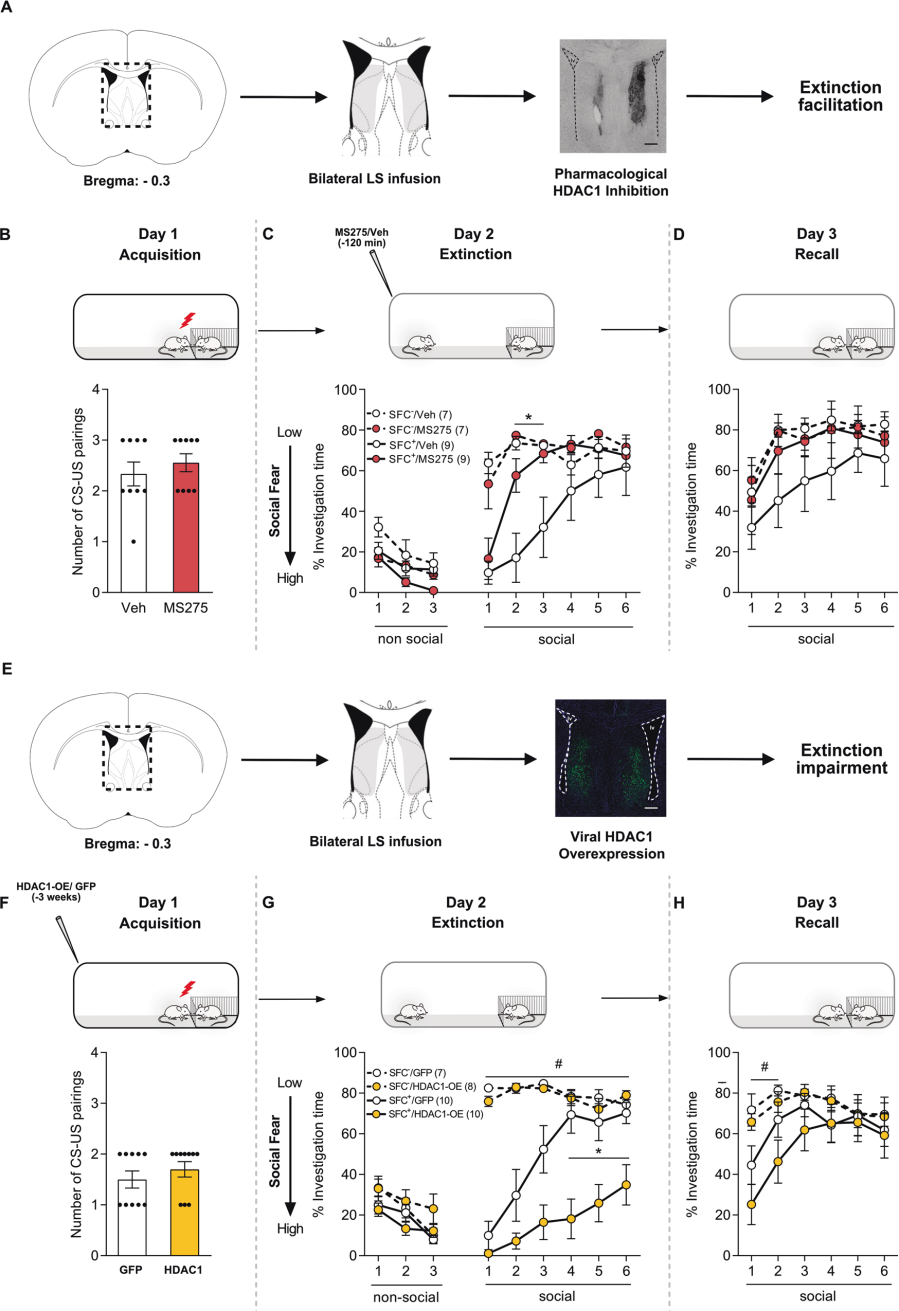
Bidirectional modulation of lateral septum (LS)-HDAC1 alters social fear extinction. Schematic representation of the lateral septum (LS), including pharmacological (**A**) and viral (**E**) interventions where Infusions within the highlighted area were considered as correct cannula placements (**A**), or correct viral infusion (**E**) site. Scalebar indicates 200 µm (**A**, **E**). Conditioned (SFC^+^) and unconditioned (SFC^−^) mice were bilaterally infused with vehicle (Veh; 0.5% DMSO in sterile Ringer; 0.2 µl/hemisphere) or MS275 (375 ng/0.2 µl/hemisphere; HDAC inhibitor) 120 min before social fear extinction (**B**–**D**) or, in separate sets of mice, with AAV-hSyn-HDAC-GFP-WPRE (HDAC1-OE; leading to HDAC1 overexpression) or AAV-hSyn-GFP-WPRE (GFP; control) 3 weeks prior to social fear acquisition (**F**–**H**). Number of CS-US pairings of SFC^+^ mice during the acquisition of social fear on day 1 (**B**, **F**). The percentage of investigation time of three non-social (empty cages) and six social (cage with a conspecific) stimuli was measured during social fear extinction (day 2; **C**, **G**) and social fear extinction recall (day 3; **D**, **H**) in SFC^−^ and SFC^+^ mice. Data represent mean CS-US pairings ± SEM (**B**, **F**) or mean percentage investigation time ± SEM (**C**, **D** and **G**, **H**). *P < 0.05 for SFC^**+**^/Veh vs. SFC^**+**^/MS275, SFC^**−**^/Veh, and SFC^**−**^/MS275 (**C**) and for SFC^+^/HDAC1-OE vs. SFC^+^/GFP (**G**); ^#^*P* < 0.05 for SFC^+^/HDAC1-OE vs. SFC^−^/GFP^,^ SFC^−^/HDAC1*-*OE (**G**, **H**).

For pharmacological blockade of LS-HDAC1, MS275 or Veh was bilaterally infused into the LS prior to extinction training leading to the following groups: SFC^+^/Veh, SFC^+^/MS275, SFC^−^/Veh, and SFC^−^/MS275 (Fig. 2A). The number of CS-US pairings did not differ among SFC^+^ groups during social fear acquisition (Fig. 2B). During social fear extinction on day 2, all SFC^+^ mice expressed high levels of social fear during the presentation of the first social stimulus, independent of their treatment, as reflected by low social investigation times. However, HDAC1 inhibition accelerated extinction, as the SFC^+^/MS275 group already displayed elevated social investigation during exposure to the 2nd and 3rd social stimulus (*P* < 0.05 vs. SFC^+^/Veh; Fig. 2C). Treatment with MS275 did not affect social investigation times in SFC^−^ mice (Fig. 2C, D). All groups showed similarly high levels of social investigation during short-term recall (Fig. 2D), indicating the successful extinction of social fear.

Second, we performed a gain-of-function experiment using AAV-mediated overexpression of HDAC1 (AAV-hSyn-HDAC1-GFP-WPRE; HDAC1-OE) or a control virus (AAV-hSyn-GFP-WPRE; GFP) within the septum of SFC^+^ and SFC^−^ mice leading to the following groups: SFC^+^/GFP, SFC^+^/HDAC-OE, SFC^−^/GFP, and SFC^−^/HDAC-OE (Fig. 2E). All groups of SFC^+^ mice received the same number of CS-US pairings during acquisition three weeks after viral transfection (Fig. 2F), suggesting that overexpression of LS-HDAC1 did not alter learning of social fear. All SFC^+^ mice independent of treatment exhibited high levels of social fear during the presentation of the first social stimulus, as reflected by reduced social investigation compared to the respective SFC^−^ groups (Fig. 2G). However, social fear extinction was significantly impaired by LS-HDAC1 overexpression, as the SFC^+^/HDAC1-OE group displayed lower social investigation during exposure of the 2nd to 6th social stimuli as opposed to the SFC^+^/GFP mice that displayed expected extinction (*P* < 0.05 vs. SFC^+^/GFP; Fig. 2G). In SFC^−^ mice, LS-HDAC1 overexpression did not affect social investigation times (Fig. 2G, H). During short-term recall, all groups of mice showed similar levels of social investigation (Fig. 2H). Overexpression of HDAC1 was confirmed by western blot analysis (Supplementary Fig. S1).

### Pre-extinction inhibition of LS-HDAC1 leads to dynamic changes in gene expression

**Fig. 3:**
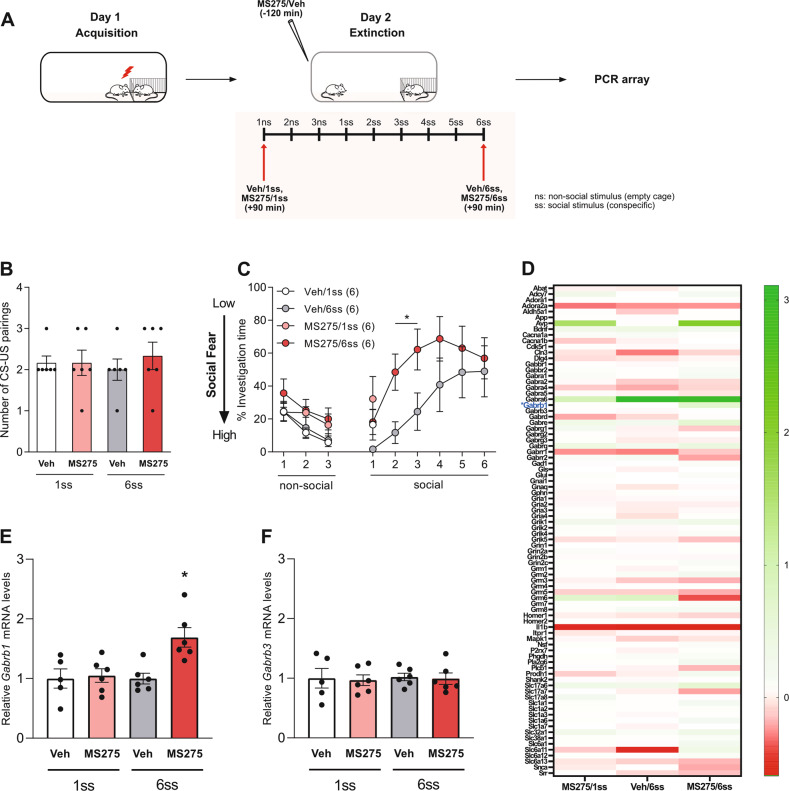
Inhibition of HDAC1 with the lateral septum (LS) leads to dynamic changes in the expression of genes related to GABA and glutamate signaling. Schematic representation of the experimental timeline wherein red arrows indicate the time point of sacrifice (**A**). Mice were fear conditioned on day 1 of the SFC paradigm. The number of CS-US pairings for all groups of conditioned (SFC^+^) mice (**B**). SFC^+^ mice were bilaterally infused with vehicle (Veh; Ringer; 0.2 µl/hemisphere) or MS275 (375 ng/0.2 µl/hemisphere) 120 min before social fear extinction (**C**). Total mRNA isolated from septum 90 min either after abridged (Veh/1ss and MS275/1ss *n* = 5–6/group) or complete (Veh/6ss and MS275/6ss; *n* = 6/group; **C**) social fear extinction was used for PCR array analysis, which assessed genes associated with GABA and glutamate signaling (normalized against Veh/1 ss; Heatmap; **D**). Relative gene expression of *Gabrb1* (**E**) and *Gabrb3* (**F**) within the LS of SFC^+^ mice treated with Veh or MS275 and exposed to 1ss or 6ss. Data represent mean CS-US pairings ± SEM (**B**), mean investigation time ± SEM (**C**), relative mRNA levels ± SEM (**E**, **F**). *P < 0.05 MS275/6ss vs. Veh/6ss and MS275/6ss vs. all other groups (**E**).

To identify the possible downstream targets of LS-HDAC1, SFC^+^ mice were bilaterally infused with either Veh or with MS275 120 min before social fear extinction and were sacrificed 90 min after exposure to either the 1st (Veh/1ss and MS275/1ss) or the 6th (Veh/6ss and MS275/6ss) social stimulus (Fig. 3A). Septal tissue micropunches were obtained and processed using a PCR array for GABA and glutamate signaling (see “Materials and methods”). All SFC^+^ groups received a similar number of CS-US pairings during social fear acquisition (Fig. 3B). During extinction, all groups of SFC^+^ mice displayed low social investigation during exposure to the 1st social stimulus. However, during extinction, SFC^+^ mice sacrificed after the 6th social stimulus showed incremental social contact with every exposure suggesting proper social fear extinction (Fig. 3C). In confirmation of results in Fig. 2C, social fear extinction was accelerated in MS275/6ss compared to Veh/6ss mice as evident from higher social investigation times during the presentation to the 2nd and 3rd social stimulus (*P* < 0.05 vs. Veh/6ss; Fig. 3C). PCR array analysis for GABA and glutamate signaling-related genes revealed dynamic changes in many genes, as shown in the heatmap (Fig. 3D). Complete PCR Array genelist along with respective fold change and *P* values is provided in Supplementary Table S3. However, one gene that was explicitly upregulated in MS275-treated mice after complete extinction training was *Gabrb1* (*P* < 0.05 vs. all other groups; Fig. 3E), while its closely related *Gabrb3* gene remained unchanged (Fig. 3F).

### Pharmacological activation of LS-GABA-A receptors facilitates extinction learning

**Fig. 4:**
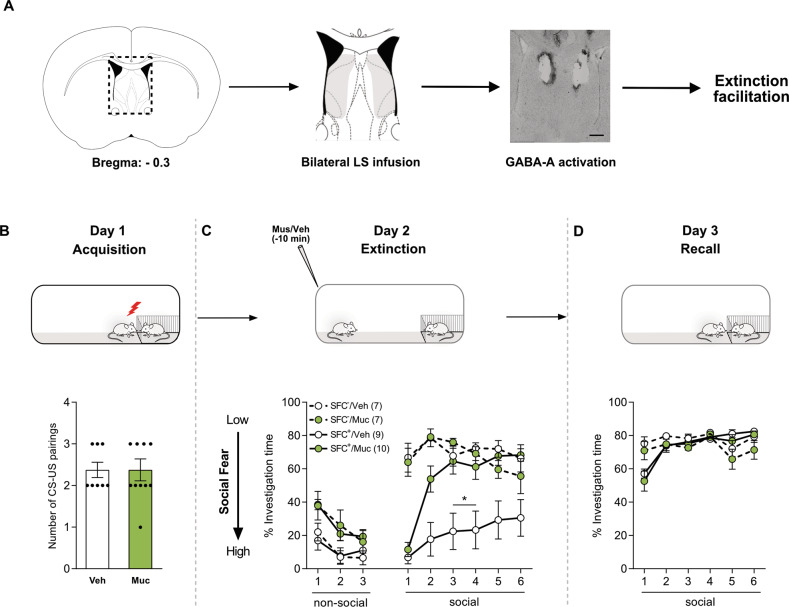
Activation of GABA-A receptors within the lateral septum (LS) facilitates social fear extinction. Schematic representation of LS, including pharmacological intervention. Scalebar indicates 200 µm and infusions within the highlighted area were considered as correct cannula placements (**A**). The number of CS-US pairings of both groups of conditioned (SFC^+^) mice during social fear acquisition on day 1 (**B**). SFC^+^ and unconditioned (SFC^−^) mice were bilaterally infused with vehicle (Veh; 0.5% DMSO in sterile Ringer; 0.2 µl/hemisphere) or Muscimol (Muc; 0.2 µg/0.2 µl/hemisphere) 10 min prior to extinction. The percentage of investigation time of three non-social (empty cages) and six social (cage with a conspecific) stimuli was measured during social fear extinction (day 2; **C**) and social fear extinction recall (day 3; **D**) in SFC^−^ and SFC^+^ mice. Data represent mean CS-US pairings ± SEM (**B**) or mean percentage investigation time ± SEM (**C**, **D**). *P < 0.05 for SFC^**+**^/Muc vs. all other groups (**C**).

Based on the results of HDAC1-inhibition-induced post-extinction elevation in *Gabrb1* expression, we hypothesized that GABAergic signaling might underly the extinction-accelerating effect of LS-HDAC inhibition. To test this idea, we locally activated the LS-GABA-A signaling by infusing Muc, a specific GABA-A agonist [25], or Veh, leading to the following groups: SFC^+^/Veh and SFC^+^/Muc, SFC^−^/Veh, and SFC^−^/Muc (Fig. 4A). SFC^+^ mice infused with either Muc or Veh required the same number of CS-US pairings to acquire social fear (Fig. 4B). During fear extinction, all SFC^+^ mice spent significantly less time investigating the social stimulus during exposure to the 1st social stimulus than their SFC^−^ counterparts (*P* < 0.05 vs. SFC^−^/Veh and SFC^−^/Muc; Fig. 4C), which pointed towards a successful acquisition of social fear. However, SFC^+^/Muc mice displayed a more rapid extinction. Specifically, they showed social investigation levels similar to that of the SFC^−^ mice, which were significantly higher than that of SFC^+^/Veh mice already while being exposed to the 2nd social stimulus (*P* < 0.05 vs. SFC^+^/Veh: Fig. 4C). During short-term recall, all groups of mice showed similar social investigation (Fig. 4D), suggesting successful extinction.

### Pharmacological inhibition of LS-HDAC1 or the activation of LS-GABA signaling leads to enduring social fear extinction

**Fig. 5:**
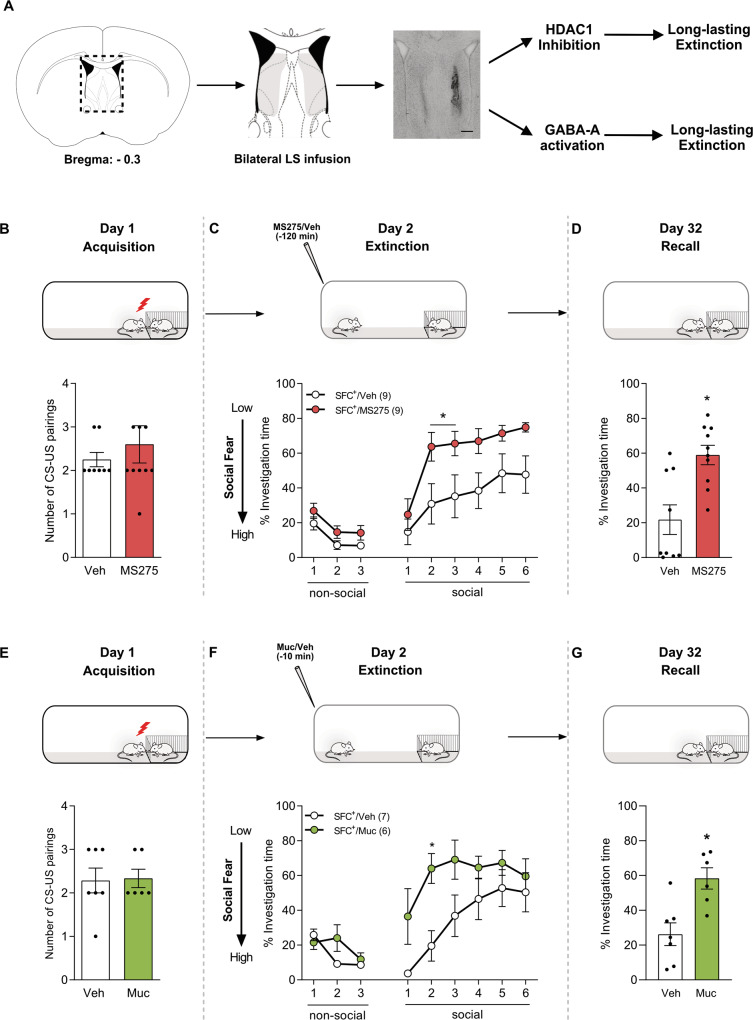
Inhibition of HDAC1 or activation of GABA-A receptors within the lateral septum (LS) enhances long-lasting social fear extinction. Schematic representation of LS, including pharmacological interventions. Scalebar indicates 200 µm and infusions within the highlighted area were considered as correct cannula placements (**A**). Inhibition of HDAC1 (**B**–**D**) and activation of GABA-A receptors (**E**–**G**) facilitated long-lasting social fear extinction. Conditioned (SFC^+^) and unconditioned (SFC^−^) mice were bilaterally infused with vehicle (Veh; 0.5% DMSO in sterile Ringer; 0.2 µl/hemisphere) or MS275 (375 ng/0.2 µl/hemisphere) (**B**–**D**) 120 min prior to extinction or in a separate cohort of mice with Veh (0.5% DMSO in sterile Ringer; 0.2 µl/hemisphere) or Muscimol (Muc; 0.2 µg/0.2 µl/hemisphere) 10 min prior to social fear extinction. Number of CS-US pairings of SFC^+^ mice during social fear acquisition on day 1 (**B**, **E**). The percentage of investigation time of three non-social (empty cages) and six social (cage with a conspecific) stimuli was measured during social fear extinction (day 2; **C**, **F**) and social fear extinction recall (day 32; **D**, **G**) in SFC^−^ and SFC^+^ mice. Data represent mean CS-US pairings ± SEM (**B**, **E**) or mean percentage investigation time ± SEM (**C**, **D** and **F**, **G**). *P < 0.05 for SFC^**+**^/Veh vs. SFC^**+**^/MS275 (**C**, **D**); *P < 0.05 for SFC^+^/Veh vs. SFC^+^/Muc (**F**, **G**).

Based on the improved extinction learning we observed both after LS-HDAC1 inhibition and LS-GABA signaling activation, we next studied the enduring nature of these pharmacological manipulations (Fig. 5A).

In the first experiment, we tested the effect of HDAC1 blockade on the formation of enduring extinction memory in SFC^+^/Veh and SFC^+^/MS275 mice. Both Veh and MS275-infused SFC^+^ mice received the same number of CS-US pairings during social fear acquisition (Fig. 5B). Confirming our previous observations (Figs. 2C and 3C), SFC^+^ mice infused with MS275 showed increased social investigation during exposure to the 2nd and 3rd social stimuli during social fear extinction training (*P* < 0.05 vs. SFC^+^/Veh; Fig. 5C). Consequently, both groups showed similar levels of social investigation times during exposure to the 6th social stimulus, suggesting successful extinction. Despite successful extinction, during long-term recall performed 30 days after extinction training, SFC^+^/MS275 mice showed significantly higher levels of social investigation (*P* < 0.05 vs. SFC^+^/Veh; Fig. 5D), demonstrating improved long-lasting extinction memory after HDAC1 inhibition.

The same experiment was performed in a separate set of SFC^+^ mice locally infused with Muc or Veh (SFC^+^/Veh and SFC^+^/Muc groups). Both groups received similar CS-US pairings indicating equal acquisition (Fig. 5E) and exhibited similarly low social investigation during exposure to the 1st social stimulus during extinction training (Fig. 5F). However, as in the previous experiment (Fig. 4C), the SFC^+^/Muc group had an accelerated social fear extinction (*P* < 0.05 vs. SFC^+^/Veh; Fig. 5F). Interestingly, during long-term recall on day 32, SFC^+^/Muc mice showed increased social investigation compared to the SFC^+^/Veh group (*P* < 0.05 vs. SFC^+^/Veh; Fig. 5G), suggesting that LS-GABA-A activation also results in enhanced long-lasting extinction memory.

## Discussion

Processes regulating the transition from short-term to long-term memory in the context of fear-related behaviors, specifically of SAD, are not well understood. In this study, using the SFC paradigm combined with pharmacological and viral approaches, we describe a novel epigenetic mechanism mediated by HDAC1 within the LS regulating the long-lasting social fear extinction memory in male mice. Specifically, we observed (i) increased activity-inducing phosphorylation of HDAC1 within the septum of SFC^+^ mice during social fear extinction, (ii) negative regulation of social fear expression by HDAC1 within the LS, (iii) an enhanced expression of *Gabrb1* in the septum in response to the combination of HDAC1 inhibition and extinction, (iv) facilitation of fear extinction by GABA-A receptor activation, and (iv) long-lasting consolidation of extinction memory after pharmacological inhibition of HDAC1 or activation of GABA-A receptors within the LS.

Class I HDACs have been previously associated with social behavior deficits [29–31] and associative learning [16, 17, 20, 32]. Considering this premise, we tested for septal HDAC1 protein dynamics during social fear extinction and found that social fear extinction in SFC^+^ mice enhanced pHDAC1 and ppHDAC1 (Fig. 1E, F), although HDAC1 protein levels remained unchanged (Fig. 1D). Casein kinase 2-mediated post-translational phosphorylations of HDAC1 at ser421 and ser423 are known to increase its catalytic (i.e., deacetylase) activity and its association with large repressor complexes, such as Sin3A and CoREST complexes [33]. Increased phosphorylation at these sites resulted from the extinction of social fear suggests that extinction training increases HDAC1 activity only in SFC^+^ mice. This enhancement was not seen in SFC^−^ mice or SFC^+^ mice that went through an abridged extinction, suggesting that complete extinction training in socially fearful mice is essential for HDAC1 phosphorylation to be increased.

We further revealed a causal link between the elevated HDAC1 activity within the LS and social fear. (i) Pharmacological blockade of LS-HDAC1 120 min prior to fear extinction facilitated extinction of social fear (Fig. 2C). (ii) Conversely, AAV-mediated overexpression of HDAC1 within the LS impaired extinction of social fear (Fig. 2G). As mentioned earlier, HDAC1 and other class I HDACs have been described as critical regulators of fear memory [16]. For example, reduced HDAC3 occupancy at the cFos promoter in the dorsal hippocampus and basolateral amygdala correlated with contextual fear memory formation without affecting cued fear [20]. In another study, RGFP963—a small molecule inhibitor of class I HDACs—enhanced consolidation of cued fear [34]. Considering that the LS is crucial for processing social cues during the formation of associative memories [12], our results suggest a novel function for LS-HDAC1 as a negative regulator of social fear extinction learning.

In order to reveal downstream targets for HDAC1, which might mediate its effect on extinction learning, we performed a PCR array for GABA and glutamate signaling-related genes in SFC^+^ mice with or without HDAC1 inhibition. We found *Gabrb1* expression to be increased in the septal tissue of mice receiving MS275 prior to extinction training (Fig. 3E). The observed change in *Gabrb1* expression was specific, as *Gabrb3*, which codes for the β3 subunit of the GABA-A receptor and is critical for natural social behavior [33], remained unchanged after LS-HDAC1 inhibition and social fear extinction (Fig. 3F). *Gabrb1* codes for the β1 subunit of the GABA-A receptor, which is essential for assembling functional GABA-A receptors [35, 36]. Although various studies have proposed differential roles for GABA-A signaling in fear extinction [37], Muc-mediated activation of GABA-A signaling with the hippocampal CA1 region enhances extinction of contextual fear [38], while similar manipulation within the infralimbic prefrontal cortex or the basolateral amygdala enhances extinction of cued fear [39]. Studies have shown that, reduction in GABA-A receptor function resulting from the downregulation of *Gabra1* expression due to reduced H3 promoter acetylation is mediated by HDAC2 and HDAC3 under conditions of chronic ethanol exposure [40]. A similar mechanism could underlie our result. Thus, HDAC1 inhibition possibly results in enhanced H3 and H4 acetylation and, consequently in enhanced *Gabrb1* expression and GABA signaling within the LS, leading to enhanced extinction learning in a social context. In confirmation of this hypothesis, activation of the LS-GABA-A receptor using Muc facilitated extinction learning similar to the observed effect of LS-HDAC1 inhibition (Fig. 4C). The LS is majorly GABAergic and is known to regulate motivated behaviors [12]. Further, the LS receives glutamatergic input from the hippocampal CA2 regions, which code for social memory [41, 42]. However, these glutamatergic inputs have a defined spatial organization and activate a specific ensemble of neurons within the LS [12, 43], leading to specific downstream behavioral effects. In the context of social fear extinction, such inputs might communicate the negative valence of the social stimulus in SFC^+^ mice, thus causing social avoidance and social fear. However, an overall inhibition of the LS caused by enhanced local GABAergic signaling is likely to disinhibit the social approach to improve social learning.

As mentioned earlier, relapse and recurrence of SAD in patients who have undergone complete remission are very high [44–46]. Thus, we tested for long-term fear extinction memory following LS-HDAC1 inhibition or LS-GABA-A activation. Notably, both HDAC1 inhibition and GABA-A receptor activation within the LS sufficiently increased the endurance of social fear extinction and prevented the recurrence of aversive social memories as found in vehicle-treated controls 30 days after social fear extinction (Fig. 5D, G). These data demonstrate that the accelerated learning acutely observed by HDAC1 inhibition and GABA-A receptor activation led to more substantial extinction memory consolidation, making it highly enduring. This corroborates the idea that HDAC inhibitors are effective cognitive enhancers [21, 47]. SAD is known to have a higher prevalence in females [48] and we have also established the SFC paradigm in female mice [11]. Moreover, there have been contrasting effects of HDAC inhibition on cognition in female mice depending on the HDAC subtype and the specific context being examined [49, 50]. Thus, one might consider studying the abovementioned mechanism in female mice. However, this examination is out of scope and should be an interesting follow-up study.

In summary, our study provides critical insights into the molecular mechanisms involved in regulating social fear extinction and implicates that HDAC1-mediated inhibition of GABAergic signaling within the LS is a negative regulator of both acute and long-term social fear extinction in mice. To the best of our knowledge, this is the first study to causally link HDAC1 to the regulation of aversive social memories. This study adds to the growing body of evidence placing HDACs as potential regulators of associative memory and specifically propounds HDAC1 as a potential therapeutic target for SAD.

## Supplementary information

Supplementary table 3

Supplementary material
