# Supplementary material for: HDAC1-mediated regulation of GABA signaling within the lateral septum facilitates long-lasting social fear extinction in male mice
Source: Transl Psychiatry. 2023 Jan 17;13:10. doi: 10.1038/s41398-023-02310-y (PMC9842607; doi:10.1038/s41398-023-02310-y)
Supplement: Supplementary file 1 — Supplementary table 3 [file 41398_2023_2310_MOESM1_ESM.docx]

| **CS-GABA-Glu PCR Array** | | | | |  |  |  |  |  |  |  |  |
| --- | --- | --- | --- | --- | --- | --- | --- | --- | --- | --- | --- | --- |
|  |  |  |  |  |  |  |  |  |  |  |  |  |
| **Gene** | **Ensemble Gene ID** | **SFC+ / Veh / 1ss** | | ***vs SFC+ / Veh / 1ss*** | | | | | | | | |
|  |  |  |  | **SFC+ / Veh /6ss** | | | **SFC+ / MS275 / 1ss** | | | **SFC+ / MS275 / 6ss** | | |
|  |  | **Fold change** | **SEM** | **Fold-change** | **SEM** | **p-value** | **Fold-change** | **SEM** | **p-value** | **Fold-change** | **SEM** | **p-value** |
| Abat | ENSMUSG00000057880 | 1 | 0.1546 | -0.0618 | 0.1347 | 0.7688 | -0.0489 | 0.0875 | 0.7801 | 0.0528 | 0.1148 | 0.7857 |
| Adcy7 | ENSMUSG00000031659 | 1 | 0.1299 | -0.0027 | 0.1576 | 0.9901 | 0.2998 | 0.1091 | 0.1085 | 0.1597 | 0.1050 | 0.3582 |
| Adora1 | ENSMUSG00000042429 | 1 | 0.1326 | 0.0214 | 0.0764 | 0.8871 | 0.0322 | 0.0912 | 0.8414 | 0.0348 | 0.0758 | 0.8168 |
| Adora2a | ENSMUSG00000020178 | 1 | 0.3271 | -0.2452 | 0.0963 | 0.4548 | -0.2853 | 0.0971 | 0.3875 | -0.2415 | 0.1493 | 0.4933 |
| Aldh5a1 | ENSMUSG00000035936 | 1 | 0.1643 | -0.1295 | 0.0916 | 0.4891 | -0.0169 | 0.0966 | 0.9284 | 0.0236 | 0.0943 | 0.8992 |
| App | ENSMUSG00000022892 | 1 | 0.1511 | -0.0106 | 0.0749 | 0.9484 | 0.0164 | 0.0588 | 0.9160 | 0.0147 | 0.0594 | 0.9247 |
| Avp | ENSMUSG00000037727 | 1 | 0.5277 | 0.0355 | 0.4901 | 0.9618 | 1.5658 | 1.6684 | 0.4324 | 2.0286 | 2.0162 | 0.3966 |
| Bdnf | ENSMUSG00000048482 | 1 | 0.1621 | 0.4044 | 0.3052 | 0.3001 | 0.2827 | 0.3494 | 0.5110 | 0.2106 | 0.3370 | 0.6116 |
| Cacna1a | ENSMUSG00000034656 | 1 | 0.0919 | 0.1199 | 0.1356 | 0.5016 | 0.0931 | 0.0963 | 0.5078 | 0.0342 | 0.0985 | 0.8084 |
| Cacna1b | ENSMUSG00000004113 | 1 | 0.1530 | -0.0493 | 0.0980 | 0.7854 | -0.1320 | 0.0848 | 0.4493 | 0.0118 | 0.1281 | 0.9539 |
| Cdk5r1 | ENSMUSG00000048895 | 1 | 0.1685 | -0.0309 | 0.0763 | 0.8629 | 0.0315 | 0.0438 | 0.8477 | -0.0251 | 0.0665 | 0.8851 |
| Cln3 | ENSMUSG00000030720 | 1 | 0.1169 | -0.2766 | 0.0405 | 0.0390 | -0.0770 | 0.0500 | 0.5342 | -0.1000 | 0.0762 | 0.4779 |
| Dlg4 | ENSMUSG00000020886 | 1 | 0.1464 | -0.0729 | 0.0896 | 0.6691 | -0.0900 | 0.0436 | 0.5381 | -0.0414 | 0.0806 | 0.8010 |
| Gabbr1 | ENSMUSG00000024462 | 1 | 0.1334 | -0.0332 | 0.0990 | 0.8430 | 0.0241 | 0.0593 | 0.8642 | 0.0624 | 0.0866 | 0.6946 |
| Gabbr2 | ENSMUSG00000039809 | 1 | 0.1183 | 0.0366 | 0.0633 | 0.7809 | 0.0660 | 0.1121 | 0.6963 | 0.1214 | 0.1909 | 0.6201 |
| Gabra1 | ENSMUSG00000010803 | 1 | 0.1225 | 0.0255 | 0.1134 | 0.8822 | 0.0836 | 0.0968 | 0.6002 | 0.1421 | 0.1388 | 0.4716 |
| Gabra2 | ENSMUSG00000000560 | 1 | 0.1878 | -0.1215 | 0.0713 | 0.5319 | -0.0335 | 0.1051 | 0.8743 | -0.0947 | 0.0854 | 0.6373 |
| Gabra4 | ENSMUSG00000029211 | 1 | 0.2849 | -0.1859 | 0.0377 | 0.4937 | -0.1267 | 0.0981 | 0.6606 | -0.1004 | 0.2048 | 0.7761 |
| Gabra5 | ENSMUSG00000055078 | 1 | 0.1509 | -0.0496 | 0.0975 | 0.7818 | -0.0224 | 0.0645 | 0.8873 | -0.0735 | 0.0904 | 0.6740 |
| Gabra6 | ENSMUSG00000020428 | 1 | 0.6337 | 3.1103 | 1.1761 | 0.1602 | 0.9757 | 0.8134 | 0.4591 | 2.9725 | 1.4304 | 0.2734 |
| Gabrb1 | ENSMUSG00000029212 | 1 | 0.1613 | -0.0016 | 0.0900 | 0.9929 | 0.0492 | 0.1133 | 0.8038 | 0.6899 | 0.1646 | 0.0159 |
| Gabrb3 | ENSMUSG00000033676 | 1 | 0.1650 | -0.0323 | 0.0892 | 0.8607 | 0.0203 | 0.0623 | 0.9041 | -0.0072 | 0.0967 | 0.9694 |
| Gabrd | ENSMUSG00000029054 | 1 | 0.2586 | -0.0930 | 0.0409 | 0.7047 | -0.1911 | 0.0750 | 0.4602 | -0.0164 | 0.2187 | 0.9621 |
| Gabre | ENSMUSG00000031340 | 1 | 0.2011 | 0.1909 | 0.2934 | 0.6198 | 0.3353 | 0.2758 | 0.3693 | 0.6327 | 0.3365 | 0.1608 |
| Gabrg1 | ENSMUSG00000001260 | 1 | 0.1654 | -0.0506 | 0.1421 | 0.8205 | 0.1512 | 0.1845 | 0.5646 | -0.1292 | 0.0664 | 0.4570 |
| Gabrg2 | ENSMUSG00000020436 | 1 | 0.1475 | -0.0428 | 0.1174 | 0.8230 | 0.0282 | 0.1048 | 0.8766 | 0.0208 | 0.0817 | 0.9002 |
| Gabrg3 | ENSMUSG00000055026 | 1 | 0.1597 | -0.0673 | 0.0807 | 0.7005 | 0.0525 | 0.1060 | 0.7840 | -0.0528 | 0.1010 | 0.7785 |
| Gabrq | ENSMUSG00000031344 | 1 | 0.2243 | 0.1976 | 0.3477 | 0.6600 | 0.4369 | 0.3328 | 0.3253 | 0.4908 | 0.2797 | 0.2168 |
| Gabrr1 | ENSMUSG00000028280 | 1 | 0.3224 | -0.2714 | 0.1131 | 0.4137 | -0.2479 | 0.1540 | 0.4809 | -0.1402 | 0.3176 | 0.7655 |
| Gabrr2 | ENSMUSG00000023267 | 1 | 0.1769 | 0.1355 | 0.1909 | 0.6209 | 0.0916 | 0.3024 | 0.8104 | -0.2125 | 0.1228 | 0.3373 |
| Gad1 | ENSMUSG00000070880 | 1 | 0.1678 | -0.0074 | 0.1260 | 0.9722 | -0.0118 | 0.0720 | 0.9464 | 0.0855 | 0.0663 | 0.6237 |
| Gls | ENSMUSG00000026103 | 1 | 0.1527 | -0.0516 | 0.0859 | 0.7650 | 0.0092 | 0.0792 | 0.9561 | -0.0010 | 0.1448 | 0.9964 |
| Glul | ENSMUSG00000026473 | 1 | 0.1616 | 0.0012 | 0.0830 | 0.9944 | 0.0614 | 0.0534 | 0.7055 | 0.0941 | 0.0828 | 0.5981 |
| Gnai1 | ENSMUSG00000057614 | 1 | 0.1396 | 0.0113 | 0.0967 | 0.9472 | 0.0147 | 0.0641 | 0.9212 | -0.0050 | 0.0450 | 0.9712 |
| Gnaq | ENSMUSG00000024639 | 1 | 0.1356 | -0.0701 | 0.0840 | 0.6591 | -0.0206 | 0.0703 | 0.8904 | -0.0319 | 0.0505 | 0.8180 |
| Gphn | ENSMUSG00000047454 | 1 | 0.1437 | 0.0381 | 0.1034 | 0.8306 | -0.0395 | 0.0584 | 0.7915 | -0.0064 | 0.0709 | 0.9672 |
| Gria1 | ENSMUSG00000020524 | 1 | 0.1392 | -0.0339 | 0.0851 | 0.8340 | -0.0382 | 0.0656 | 0.7981 | -0.0211 | 0.0545 | 0.8828 |
| Gria2 | ENSMUSG00000033981 | 1 | 0.1537 | -0.0599 | 0.0996 | 0.7428 | -0.0285 | 0.0588 | 0.8565 | -0.0540 | 0.0950 | 0.7633 |
| Gria3 | ENSMUSG00000001986 | 1 | 0.1559 | -0.0622 | 0.0819 | 0.7186 | 0.0333 | 0.0449 | 0.8286 | -0.0343 | 0.1749 | 0.8891 |
| Gria4 | ENSMUSG00000025892 | 1 | 0.1580 | -0.0800 | 0.0923 | 0.6593 | 0.0622 | 0.1628 | 0.7927 | 0.0460 | 0.1538 | 0.8406 |
| Grik1 | ENSMUSG00000022935 | 1 | 0.1397 | 0.1906 | 0.2038 | 0.4792 | 0.2702 | 0.1181 | 0.1708 | 0.3218 | 0.1552 | 0.1650 |
| Grik2 | ENSMUSG00000056073 | 1 | 0.1751 | -0.0328 | 0.1052 | 0.8710 | 0.0612 | 0.1084 | 0.7647 | 0.0333 | 0.0395 | 0.8434 |
| Grik4 | ENSMUSG00000032017 | 1 | 0.1217 | -0.0401 | 0.1096 | 0.8118 | 0.0510 | 0.0581 | 0.6980 | 0.0930 | 0.0848 | 0.5359 |
| Grik5 | ENSMUSG00000003378 | 1 | 0.1502 | -0.0646 | 0.0937 | 0.7139 | -0.0829 | 0.1525 | 0.7105 | -0.1509 | 0.0990 | 0.4088 |
| Grin1 | ENSMUSG00000026959 | 1 | 0.1425 | -0.0134 | 0.0895 | 0.9358 | 0.0068 | 0.0750 | 0.9654 | 0.0297 | 0.0575 | 0.8407 |
| Grin2a | ENSMUSG00000059003 | 1 | 0.1533 | -0.0126 | 0.0945 | 0.9437 | 0.0752 | 0.0911 | 0.6707 | 0.0392 | 0.2349 | 0.8969 |
| Grin2b | ENSMUSG00000030209 | 1 | 0.1135 | -0.0348 | 0.0873 | 0.8104 | -0.0199 | 0.0594 | 0.8736 | -0.0291 | 0.1382 | 0.8777 |
| Grin2c | ENSMUSG00000020734 | 1 | 0.1969 | 0.0436 | 0.1216 | 0.8494 | 0.0421 | 0.0354 | 0.8222 | 0.1139 | 0.1336 | 0.6340 |
| Grm1 | ENSMUSG00000019828 | 1 | 0.1918 | -0.0469 | 0.1025 | 0.8258 | 0.0321 | 0.1499 | 0.8963 | 0.0664 | 0.1699 | 0.8006 |
| Grm2 | ENSMUSG00000023192 | 1 | 0.2373 | 0.1040 | 0.0818 | 0.6652 | 0.0188 | 0.1696 | 0.9488 | 0.1866 | 0.3027 | 0.6498 |
| Grm3 | ENSMUSG00000003974 | 1 | 0.2008 | -0.1414 | 0.0612 | 0.4834 | -0.0492 | 0.0649 | 0.8063 | -0.1726 | 0.1002 | 0.4376 |
| Grm4 | ENSMUSG00000063239 | 1 | 0.1864 | 0.0228 | 0.0339 | 0.8977 | -0.0262 | 0.1043 | 0.9005 | 0.0414 | 0.1462 | 0.8632 |
| Grm5 | ENSMUSG00000049583 | 1 | 0.1613 | -0.1104 | 0.0859 | 0.5412 | -0.1133 | 0.0785 | 0.5207 | -0.1796 | 0.0656 | 0.2982 |
| Grm6 | ENSMUSG00000000617 | 1 | 0.6649 | 0.8226 | 0.7663 | 0.4485 | 0.7359 | 0.7811 | 0.4935 | -0.3629 | 0.1415 | 0.6080 |
| Grm7 | ENSMUSG00000056755 | 1 | 0.1559 | 0.0335 | 0.0871 | 0.8487 | 0.0410 | 0.0773 | 0.8087 | 0.0934 | 0.0830 | 0.5917 |
| Grm8 | ENSMUSG00000024211 | 1 | 0.0979 | 0.1386 | 0.1333 | 0.4409 | 0.0541 | 0.0975 | 0.7069 | 0.1256 | 0.1255 | 0.4649 |
| Homer1 | ENSMUSG00000007617 | 1 | 0.2491 | -0.0751 | 0.0837 | 0.7643 | -0.0559 | 0.0755 | 0.8210 | -0.0985 | 0.1164 | 0.7125 |
| Homer2 | ENSMUSG00000025813 | 1 | 0.1240 | 0.0276 | 0.1036 | 0.8672 | 0.0275 | 0.0622 | 0.8391 | -0.0127 | 0.0670 | 0.9269 |
| Il1b | ENSMUSG00000027398 | 1 | 0.5881 | -0.5919 | 0.1004 | 0.3035 | -0.4869 | 0.0807 | 0.3894 | -0.5397 | 0.1543 | 0.3584 |
| Itpr1 | ENSMUSG00000030102 | 1 | 0.2542 | -0.0358 | 0.0775 | 0.8871 | -0.0718 | 0.0314 | 0.7642 | -0.0419 | 0.1503 | 0.8856 |
| Mapk1 | ENSMUSG00000063358 | 1 | 0.1408 | -0.1162 | 0.0731 | 0.4601 | -0.0362 | 0.0466 | 0.7976 | -0.0721 | 0.0255 | 0.5935 |
| Nsf | ENSMUSG00000034187 | 1 | 0.1216 | 0.0334 | 0.0873 | 0.8243 | 0.0453 | 0.0684 | 0.7419 | 0.0765 | 0.0715 | 0.5856 |
| P2rx7 | ENSMUSG00000029468 | 1 | 0.0535 | -0.0489 | 0.1145 | 0.7264 | 0.0065 | 0.0319 | 0.9159 | 0.0542 | 0.0676 | 0.5578 |
| Phgdh | ENSMUSG00000053398 | 1 | 0.1566 | 0.0884 | 0.0783 | 0.6066 | 0.0461 | 0.0606 | 0.7748 | 0.1049 | 0.1699 | 0.6659 |
| Pla2g6 | ENSMUSG00000042632 | 1 | 0.0905 | 0.0503 | 0.0937 | 0.7120 | 0.0350 | 0.0777 | 0.7744 | 0.2023 | 0.0848 | 0.1380 |
| Plcb1 | ENSMUSG00000051177 | 1 | 0.2231 | -0.0463 | 0.0891 | 0.8411 | -0.0139 | 0.0317 | 0.9474 | -0.1649 | 0.1235 | 0.5148 |
| Prodh1 | ENSMUSG00000003526 | 1 | 0.2276 | -0.0567 | 0.0382 | 0.7930 | -0.1143 | 0.0489 | 0.6035 | 0.1501 | 0.1481 | 0.5818 |
| Shank2 | ENSMUSG00000037541 | 1 | 0.1387 | -0.0258 | 0.0997 | 0.8803 | -0.0138 | 0.0761 | 0.9294 | 0.0166 | 0.0985 | 0.9224 |
| Slc17a6 | ENSMUSG00000030500 | 1 | 0.3832 | 0.2865 | 0.1327 | 0.4999 | 0.3191 | 0.4571 | 0.6148 | 0.6118 | 0.4893 | 0.3655 |
| Slc17a7 | ENSMUSG00000070570 | 1 | 0.2246 | -0.0576 | 0.2905 | 0.8829 | -0.0053 | 0.3905 | 0.9914 | -0.2124 | 0.3590 | 0.6450 |
| Slc17a8 | ENSMUSG00000019935 | 1 | 0.1918 | 0.0180 | 0.1789 | 0.9470 | 0.1955 | 0.1500 | 0.4359 | -0.0111 | 0.0511 | 0.9567 |
| Slc1a1 | ENSMUSG00000024935 | 1 | 0.1263 | 0.0293 | 0.0895 | 0.8502 | 0.0661 | 0.0276 | 0.5889 | 0.1602 | 0.0462 | 0.2318 |
| Slc1a2 | ENSMUSG00000005089 | 1 | 0.1448 | 0.0340 | 0.0675 | 0.8265 | 0.0457 | 0.0379 | 0.7469 | 0.0656 | 0.0865 | 0.6950 |
| Slc1a3 | ENSMUSG00000005360 | 1 | 0.0686 | -0.0283 | 0.0828 | 0.8038 | 0.0122 | 0.0367 | 0.8723 | 0.0199 | 0.0536 | 0.8217 |
| Slc1a6 | ENSMUSG00000005357 | 1 | 0.1701 | -0.0078 | 0.1681 | 0.9749 | 0.0502 | 0.1350 | 0.8199 | 0.1653 | 0.1850 | 0.5343 |
| Slc1a7 | ENSMUSG00000008932 | 1 | 0.1363 | -0.0480 | 0.1486 | 0.8205 | 0.0121 | 0.1016 | 0.9438 | 0.0104 | 0.1100 | 0.9532 |
| Slc32a1 | ENSMUSG00000037771 | 1 | 0.0949 | 0.1043 | 0.1175 | 0.5198 | 0.2907 | 0.1398 | 0.1344 | 0.3181 | 0.1401 | 0.1059 |
| Slc38a1 | ENSMUSG00000023169 | 1 | 0.1226 | 0.0135 | 0.1293 | 0.9423 | 0.0520 | 0.0757 | 0.7165 | 0.1007 | 0.1026 | 0.5409 |
| Slc6a1 | ENSMUSG00000030310 | 1 | 0.1664 | -0.0118 | 0.1628 | 0.9608 | 0.0502 | 0.1585 | 0.8328 | 0.2100 | 0.2183 | 0.4788 |
| Slc6a11 | ENSMUSG00000030307 | 1 | 0.1426 | -0.4587 | 0.1229 | 0.0367 | -0.1259 | 0.1207 | 0.5141 | 0.3562 | 0.6393 | 0.6319 |
| Slc6a12 | ENSMUSG00000030109 | 1 | 0.1426 | -0.0028 | 0.0602 | 0.9852 | 0.0550 | 0.1113 | 0.7643 | 0.1082 | 0.2393 | 0.7077 |
| Slc6a13 | ENSMUSG00000030108 | 1 | 0.2151 | -0.1111 | 0.0993 | 0.6303 | -0.0742 | 0.1099 | 0.7533 | -0.1688 | 0.0288 | 0.4591 |
| Snca | ENSMUSG00000025889 | 1 | 0.1757 | 0.0292 | 0.1305 | 0.8945 | -0.0634 | 0.0646 | 0.7237 | -0.1926 | 0.0192 | 0.3076 |
| Srr | ENSMUSG00000001323 | 1 | 0.1505 | -0.0958 | 0.0888 | 0.5815 | 0.0238 | 0.0365 | 0.8704 | -0.1513 | 0.0167 | 0.3470 |
