# Supplementary material for: HDAC1-mediated regulation of GABA signaling within the lateral septum facilitates long-lasting social fear extinction in male mice
Source: Transl Psychiatry. 2023 Jan 17;13:10. doi: 10.1038/s41398-023-02310-y (PMC9842607; doi:10.1038/s41398-023-02310-y)
Supplement: Supplementary file 2 — Supplementary material [file 41398_2023_2310_MOESM2_ESM.docx]

**Supplementary Information:**

**Supplementary methods:**

**Table S1: List of housekeepers used for PCR array**

| **No** | **Gene symbol** | **Gene name** |
| --- | --- | --- |
| 1 | *Actb* | Actin Beta |
| 2 | *B2m* | Beta-2-Microglobulin |
| 3 | *Gapdh* | Glyceraldehyde-3-Phosphate Dehydrogenase |
| 4 | *Gusb* | Glucuronidase Beta |
| 5 | *Hsp90ab1* | Heat Shock Protein 90 Alpha Family Class B Member 1 |

**Table S2: Statistics table**

| **Activity inducing HDAC1 phosphorylations are upregulated in response to social fear extinction in the septum of SFC^+^ mice (Figure 1).** | | |
| --- | --- | --- |
| *SFC*  *(B: Student’s T-test; C: 2-way ANOVA)*  Social fear acquisition (B)  Social fear extinction:  1ss (C)  1ss-6ss (C)  *Western Blot*  *(D-F: 1-way ANOVA)*  HDAC1 (D)  pHDAC1 (E)  ppHDAC1 (F) | *Group effect (SFC)*  T(17) = 0.9732, p = 0.4015  F(1, 17) = 26.99, p < 0.0001*  F(1, 16) = 43.96, p < 0.0001*  *Group effect (SFC x extinction)*  F(3, 31) = 0.7825, p = 0.5128  F(3, 33) = 3.842, p = 0.0183*  F(3, 32) = 4.114, p = 0.0141* | *Group x extinction effect*  F(3, 51) = 41.90, p < 0.0001*  F(8, 128) = 11.47, p < 0.0001* |
| **Bidirectional modulation of LS-HDAC1 regulates learning of social fear extinction (Figure 2).** | | |
| *SFC (MS275 vs Veh)*  *(B: Student’s T-test; C-D: 2-way ANOVA)*  Social fear acquisition (B)  Social fear extinction (C)  Social fear recall (D)  *SFC (HDAC1 vs GFP)*  *(E: Student’s T-test; F-G: 2-way ANOVA)*  Social fear acquisition (E)  Social fear extinction (F)  Social fear recall (G) | *Group effect (Treatment x SFC)*  T(16) = 0.9177, p = 0.3724  F(3, 234) = 17.30, p < 0.0001*  F(3, 156) = 5.268, p = 0.0017*  *Group effect (Treatment x SFC)*  T(18) = 0.8847, p = 0. 3880  F(3, 30) = 45.40, p < 0.0001*  F(3, 31) = 2.941, p = 0.0484* | *Group x extinction effect*  F(24, 234) = 2.619, p = 0.0001*  F(15, 156) = 0.1601, p = 0.9999  *Group x extinction effect*  F(24, 240) = 8.131, p < 0.0001*  F(15, 155) = 1.129, p = 0.3348 |
| **Pre-extinction inhibition of LS-HDAC1 led to dynamic changes in gene expression (Figure 3).** | | |
| *SFC (MS275 vs Veh)*  *(B: Student’s T-test; C: 2-way ANOVA)*  Social fear acquisition (B)  Social fear extinction:  1ss (C)  1ss-6ss (C)  *PCR array analysis*  *(E-F: 1-way ANOVA)*  *Gabrb1* (E)  *Gabrb3* (F) | *Group effect (Treatment)*  F(3, 20) = 0.247, p = 0.8625  F(1, 5) = 4.306, p = 0.0926  F(1, 5) = 9.963, p = 0.0252*  *Group effect (SFC x extinction)*  F(3, 19) = 6.519, p = 0.0032*  F(3, 19) = 0.0454, p = 0.9867 | *Treatment x extinction effect*  F(3, 15) = 0.2974, p = 0.8267  F(8, 40) = 1.42, p = 0.2181 |
| **Pharmacological activation of LS-GABA-A receptors facilitates extinction learning (Figure 4).** | | |
| *SFC (Muc vs Veh)*  *(B: Student’s T-test;*  *C-D: 2-way ANOVA)*  Social fear acquisition (B)  Social fear extinction (C)  Social fear recall (D) | *Group effect (Treatment x SFC)*  T(14) = 0, p > 0.9999  F(3, 28) = 14.54, p < 0.0001*  F(3, 30) = 1.569, p = 0.2173 | *Group x extinction effect*  F(24, 220) = 5.268, p < 0.0001*  F(15, 150) = 2.830, p = 0.0007* |
| **Pharmacological inhibition of LS-HDAC1 or the activation of LS-GABA signaling leads to enduring social fear extinction (Figure 5).** | | |
| *SFC (MS275 vs Veh)*  *(B, D: Student’s T-test; C: 2-way ANOVA)*  Social fear acquisition (B)  Social fear extinction (C)  Social fear recall (D)  *SFC (Muc vs Veh)*  *(E, G: Student’s T-test; F: 2-way ANOVA)*  Social fear acquisition (E)  Social fear extinction (F)  Social fear recall (G) | *Group effect (SFC)*  T(16) = 0.6976, p = 0.4954  F(1, 16) = 6.892, p = 0.0184*  T(16) = 2.534, p = 0.0214*  *Group effect (SFC)*  T(11) = 0.1301, p = 0.8988  F(1, 11) = 15.58, p = 0.0425*  T(11) = 3.538, p = 0.0047* | *Group x extinction effect*  F(8, 128) = 1.767, p = 0.0895  *Group x extinction effect*  F(8, 88) = 2.458, p = 0.0189* |
| **AAV mediated genetic manipulation leads to overexpression of HDAC1 (Figure S1)** | | |
| *Western blot*  *(B: Student’s T-test)*  HDAC1 overexpression (B) | *Group effect (GFP vs HDAC1)*  T(32) = 4.243, p = 0.0002* |  |

**Note: Supplementary table 3 has been separately uploaded.**

**Supplementary results:**

**AAV mediated genetic manipulation leads to overexpression of HDAC1**

**
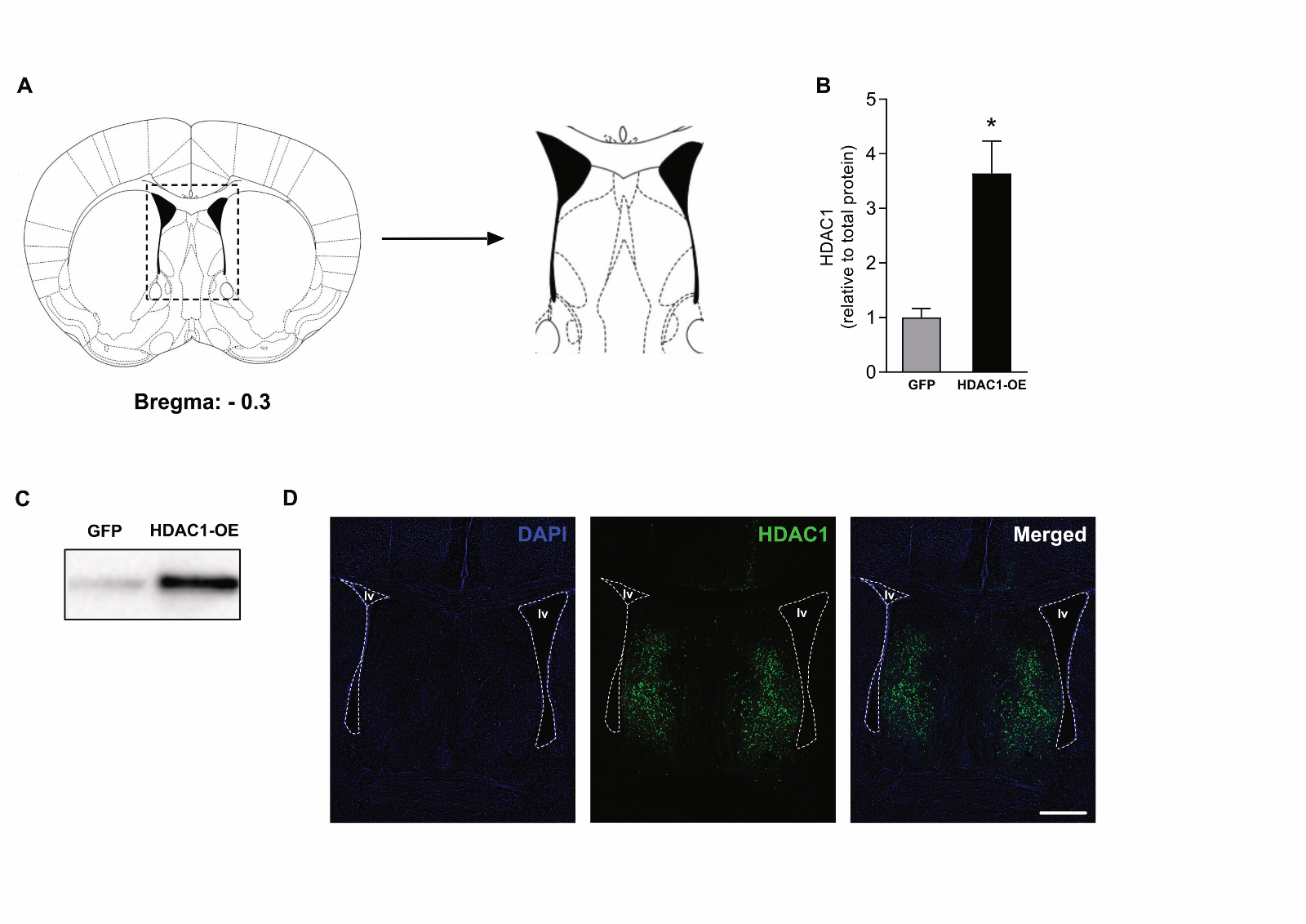
**

**Figure S1:** Genetic manipulation leads to overexpression of HDAC1. Schematic representation of the septum including viral manipulations (A). The highlighted area was considered as adequate infusion localization. Male mice were injected with AAV-hSyn-HDAC-GFP-WPRE (AAV) or AAV-hSyn-GFP-WPRE (C-AAV; control) 3 weeks prior to acquisition of social fear. After the behavioral analysis using the SFC paradigm, HDAC1 protein levels were measured from septum micropunches using Western blot (B). Representative western blot images for HDAC1 (C). Panel showing viral transfection for HDAC1 overexpression with GFP as a reporter. Scalebar indicates a length of 200µm (D). Data represent mean fold change + SEM (D-F). *p<0.05 AAV vs C-AAV (B).
